# Focal iron sparing mimicking liver lesion in post-transfusion iron overload: A case report

**DOI:** 10.1016/j.radcr.2025.07.024

**Published:** 2025-08-09

**Authors:** Lukas Endrös, Felix Hesse, Rickmer F. Braren, Lisa Steinhelfer

**Affiliations:** a Department of Radiology, School of Medicine and Health, Technical University of Munich, TUM Klinikum Rechts der Isar, Ismaninger Str. 22, 81675 Munich, Germany; b Department of Internal Medicine II, School of Medicine and Health, Technical University of Munich, TUM Klinikum Rechts der Isar, Munich, Germany; c German Cancer Consortium (DKTK), partner site Munich, a partnership between DKFZ and School of Medicine and Health, Technical University of Munich, Munich, Germany

**Keywords:** Liver MRI, MRI pitfalls, Focal iron sparing, Iron overload, Pseudolesion, Case report

## Abstract

Iron sparing, a rare imaging phenomenon in the context of hepatic iron overload, can mimic true liver lesions and pose significant diagnostic challenges—especially in oncologic patients with a history of frequent blood transfusions. We present the case of a 68-year-old woman with metastatic breast cancer and transfusion-dependent anemia who developed hepatic iron overload following multiple red blood cell transfusions. MRI showed a hyperintense lesion in segment 4b on a fat-suppressed Dixon sequence, raising initial concern for pathology. T2* mapping and chemical shift imaging revealed hepatic iron overload with relative sparing in segment 4, characterized by a marked signal drop on in-phase imaging and reduced T2* relaxation time in the surrounding liver parenchyma; the spared region itself showed a comparatively longer T2* time, consistent with focal iron sparing rather than a true lesion. Additionally, an aberrant right gastric vein supplying this region suggested altered perfusion as a possible underlying mechanism. This case underscores the importance of identifying focal iron sparing to prevent misdiagnosis and avoid unwarranted interventions.

## Introduction

Iron overload in the form of hemochromatosis can be either hereditary—most commonly caused by HFE gene mutations—or secondary to conditions such as ineffective erythropoiesis (eg, thalassemias), chronic liver disease, increased dietary iron absorption, or frequent red blood cell transfusions. Excessive iron deposition in the liver can lead to hepatic fibrosis, cirrhosis, and an increased risk of hepatocellular carcinoma (HCC). In addition, endocrine disorders such as diabetes mellitus have been associated with systemic iron overload [[1], [2], [3], [4]].

While MRI features of diffuse and focal hepatic siderosis are well established [5,6], focal iron sparing remains an underrecognized phenomenon, with only limited case reports available in the literature. Only a few studies, such as those by İdilman et al. [6], Karçaaltıncaba et al. [7], and Pecorelli et al. [8], have addressed this finding. Focal iron sparing can mimic focal liver lesions, potentially leading to misinterpretation that may result in inappropriate treatment decisions or unnecessary invasive investigations, such as biopsies.

Oncologic patients have a heightened risk of secondary iron overload because they often require frequent blood transfusions. This is mainly due to anemia caused by the underlying malignancy, bone marrow involvement, myelosuppressive chemotherapy or radiation, and surgical blood loss [9,10]. Therefore, early detection and accurate diagnosis are crucial for appropriate management and the prevention of long-term complications.

In this case report, we describe a patient with metastatic breast cancer who developed hepatic iron overload following transfusions. This illustrates the diagnostic challenge of distinguishing focal iron sparing from breast cancer metastases in the setting of secondary transfusion-related iron overload and demonstrates how altered venous perfusion can result in a hepatic pseudolesion on MRI.

## Patient history

**Fig. 1:**
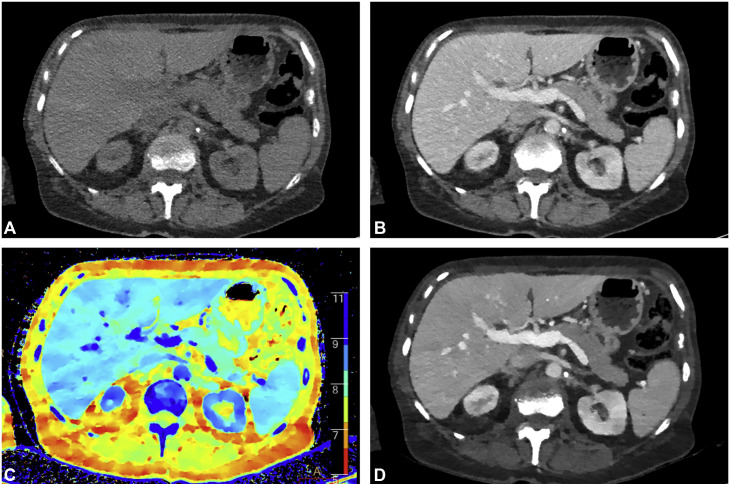
Iodine contrast-enhanced computed tomography (CT) performed on Philips IQon spectral CT with (A) virtual noncontrast, (B) portal venous contrast phase, (C) Z-effective map visualizing effective atomic number, and (D) virtual mono-energetic CT at 40 keV accentuating iodine-enhanced structures.

**Fig. 2:**
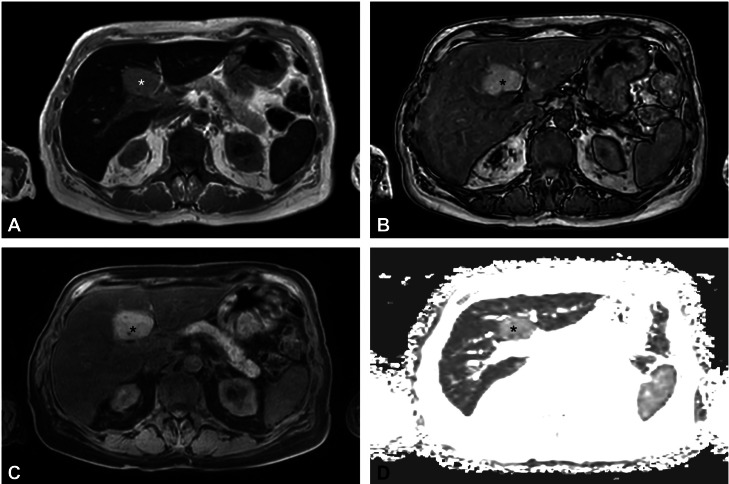
Focal iron sparing in segment 4 (*). (A) In phase and (B) opposed phase image showing signal drop on of the liver parenchyma in phase, while the lesion itself is showing relatively less signal drop. (C) T1 hyperintense lesion in fat-suppressed mDixon sequence. (D) mDixon Quant T2*-map demonstrating higher T2* relaxation time of the lesion compared to the residual liver parenchyma, indicating focal iron sparing.

A 68-year-old Caucasian female patient with locally advanced breast cancer, complicated by pulmonary and diffuse bone metastases, presented with a markedly reduced general condition. Blood test revealed megaloblastic anemia (RBC 1.8 × 10^12^/L, Hb 6.3 g/dL, MCH 35 pg, MCV 107 fl), and subsequent bone marrow aspiration confirmed metastatic infiltration consistent with disseminated bone marrow carcinomatosis. Over approximately 15 months, the patient received a total of 12 red blood cell transfusions and 5 platelet transfusions. Regular iodine-enhanced computed tomography (CT) staging examinations showed no evidence of intraparenchymal lesions (see Fig. 1). However, complementary abdominal magnetic resonance imaging (MRI) using a standard tumor protocol, performed 4 weeks after the patient’s last transfusion, demonstrated hepatic iron overload with altered signal characteristics in liver segment 4 as shown in Fig. 2.

## Imaging findings

MRI examinations were performed on a 3 Tesla (T) MRI system (Ingenia, Philips Healthcare, Amsterdam, The Netherlands) using coronar and axial T2-weighted TSE-sequences, an axial diffusion-weighted EPI-sequence with ADC-map (b: 0, 50, 300, 600, and 800 s/mm^2^), and axial T1-weighted Dixon native and dynamic Gd-enhanced (Dotagraf 0,5 mmol/mL) sequences. MRI revealed a large focal hyperintense area in the posterior area of segment 4b on the Dixon fat-suppressed Dixon sequence. Quantitative analysis using the manufacturer’s mDixon Quant software revealed hepatic steatosis with 9% fat content, with no significant difference in fat composition between the lesion and the surrounding tissue. However, the T2* map revealed an increased intralesional T2* relaxation time within the lesion (3.4 ms) compared to the adjacent liver parenchyma (1.4 ms). In-phase imaging showed a marked signal drop in the surrounding liver parenchyma but only a mild signal drop within the lesion itself, consistent with significant iron overload and relative iron sparing in segment 4 (see Fig. 2). No signal drop was observed on opposed-phase images (Table 1).

**Table 1:** Characteristics of focal signal abnormality in segment 4.

Imaging sequence	Signal characteristics
T1-weighted	Hyperintense
T2-weighted	Isointense
In-phase T1-weighted	Mild signal drop in lesion, marked signal drop in surrounding parenchyma
Out-of-phase T1-weighted	No signal drop in lesion or surrounding parenchyma
Diffusion-weighted (DWI)	No diffusion restriction
Dynamic Gd-enhanced T1-weighted	No dynamic in contrast enhancement
T2* relaxation time of segment 4 lesion vs surrounding parenchyma	3.4 vs 1.4 ms
Fat Fraction of segment 4 lesion vs surrounding parenchyma	8% vs 9%

**Fig. 3:**
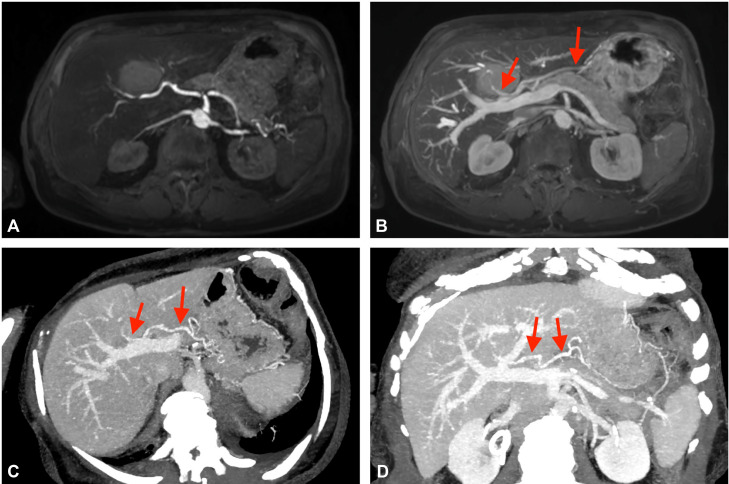
Dixon sequence in (A) arterial and (B) portal venous phase showing accessory perfusion of posterior segment 4 by an aberrant right gastric vein (*arrows*). (C) + (D) Different multiplanar reformations (MPRs) of the aberrant right gastric vein in a contrast-enhanced CT in portal venous phase (*arrows*). The vein originates from the lesser curvature of the stomach and directly supplies segment 4 of the liver. Note the different contrast enhancement between the aberrant venous vessel and the arterial vessels (ie, the celiac trunk and the common hepatic artery).

The lesion appeared isointense on T2-weighted images and showed no diffusion restriction in diffusion-weighted sequences. In dynamic gadolinium-enhanced T1-weighted images, the lesion remained hyperintense throughout all phases, without specific contrast dynamics, particularly lacking washout. Notably, during the portal venous phase, an additional vascular supply to segment 4 via an aberrant right gastric vein was detected, as demonstrated in Fig. 3. Structurally, the lesion showed no signs of invasive growth, with blood vessels passing through without obstruction or displacement.

## Discussion

In routine abdominal MRI scans, chemical shift imaging, commonly used for fat detection, can also play an important role in identifying iron deposition by demonstrating a signal drop on in-phase images [6,11]. R2 and R2* relaxometry, established methods for quantifying liver iron concentration [12,13], further validate the findings: T2 and T2* values are inversely related to iron concentration, while R2 and R2* (1000/T2 or T2*) increase linearly with iron levels [[13], [14], [15]]. Areas of focal iron sparing, most commonly found in the posterior aspect of segment 4b, may appear as pseudolesions on imaging and often correlate with regions of focal fat sparing or overload [6,7]. Focal fat sparing is generally attributed to altered perfusion due to third inflow from aberrant veins, including Sappey veins, right and left gastric veins, the gastroduodenal vein, and the pancreaticoduodenal vein [[16], [17], [18]]. Our findings support this hypothesis by demonstrating accessory perfusion of segment 4 via an aberrant right gastric vein.

Although increased iron content can also be detected on CT, often reflected as elevated density in native CT (≥72 HU) [19], its interpretation may be complicated by confounding factors such as hepatic steatosis or the deposition of other metals (eg, such as gold) [20]. Our case suggests that MRI is more sensitive in detecting both iron overload and focal variations in hepatic iron content.

Focal regions of iron sparing typically appear T1 hyperintense, retaining the normal signal intensity of the liver parenchyma compared to the surrounding iron-overloaded tissue. This pattern typically presents with a signal drop of the surrounding parenchyma on in-phase images. In contrast, focal fat deposition shows a signal drop within the lesion on opposed-phase imaging, while focal fat sparing is marked by a signal drop in the surrounding parenchyma [[6], [7], [8],^21^]. Differentiating focal iron sparing from coexisting focal fat sparing can be challenging; in such cases, combined assessment of fat fraction and R2*-relaxometry may assist in characterization [7].

Accurate classification of liver lesions in oncologic patients poses a diagnostic challenge, particularly when differentiating metastases from benign findings. Metastases can also appear T1 hyperintense—similar to the appearance of focal iron sparing—when associated with high protein content, necrosis, melanin, or methemoglobin [22]; however, they most commonly present as T1 hypointense and T2 iso- to hyperintense lesions [23]. In comparison to metastases, the benign findings of focal iron sparing or focal fat deposition generally do not produce a mass effect on vessels or other adjacent liver structures [21,24]. Additionally, metastases often show altered vascularization, resulting in distinct contrast enhancement patterns on gadolinium-enhanced imaging. For instance, while breast cancer metastases are usually hypovascular, they can occasionally appear hypervascular [25]. Diffusion-weighted imaging can also provide additional diagnostic value, as malignant lesions typically exhibit restricted diffusion due to high cellular density and reduced extracellular space [26].

Early and accurate detection of hepatic iron overload is essential, as it can elevate the risk of cirrhosis and HCC [3]. Incidental findings of pseudolesions with iron sparing can aid in diagnosis; however, differentiating them from HCC remains critical, as HCCs arising in cirrhotic livers due to hepatic iron overload typically lack iron deposition within tumor cells [27], and typically appear T1 and T2 hyperintense [8]. In clinical practice, differentiation can be achieved using LI-RADS criteria, which include key imaging features such as arterial phase hyperenhancement, delayed phase nonperipheral washout, presence of an enhancing capsule, and threshold growth on follow-up imaging [28,29].

In our case, subsequent blood tests revealed significantly elevated ferritin levels (1460 ng/mL) and increased transferrin saturation (51%). Genetic testing for hereditary hemochromatosis was negative. The lesion remained stable in size and signal characteristics on serial imaging. Although histopathologic confirmation was not pursued, the clinical context, laboratory parameters, and consistent imaging findings over time strongly support the diagnosis of benign focal iron sparing in the setting of transfusion-related secondary iron overload.

Key learning points from this case include the importance of recognizing focal iron sparing as a potential diagnostic pitfall in liver imaging, particularly in the context of aberrant venous supply. Advanced MRI techniques, such as chemical shift imaging and R2*-relaxometry, play an important role in detecting and quantifying iron deposition. Reliable differentiation from malignant lesions requires careful assessment of diffusion characteristics, contrast enhancement behavior, and the absence of mass effect on adjacent hepatic structures. In cases where HCC is considered, the use of LI-RADS criteria can further support diagnostic confidence.

## Conclusion

Increased awareness of the rare phenomenon of focal iron sparing is essential to avoid misdiagnosis, minimize unnecessary interventions, and ensure appropriate patient management.

## Patient consent

Written informed consent was obtained from the patient for the publication of this case report and accompanying images.
